# *Sclerotinia homoeocarpa* Overwinters in Turfgrass and Is Present in Commercial Seed

**DOI:** 10.1371/journal.pone.0110897

**Published:** 2014-10-21

**Authors:** Renée A. Rioux, Jeanette Shultz, Michelle Garcia, David Kyle Willis, Michael Casler, Stacy Bonos, Damon Smith, James Kerns

**Affiliations:** 1 Department of Plant of Plant Path Pathology, University of Wisconsin-Madison, Madison, Wisconsin, United States of America; 2 NewLeaf Symbiotics, BRDG Park, St. Louis, Missouri, United States of America; 3 Department of Neuroscience, University of Wisconsin-Madison, Madison, Wisconsin, United States of America; 4 Department of Biological Sciences, University of Texas El-Paso, El Paso, Texas, United States of America; 5 USDA-ARS, U.S. Dairy Forage Research Center, Madison, Wisconsin, United States of America; 6 Department of Plant Science, Rutgers University, New Brunswick, New Jersey, United States of America; 7 Department of Plant Pathology, North Carolina State University, Raleigh, North Carolina, United States of America; University of Nebraska-Lincoln, United States of America

## Abstract

Dollar spot is the most economically important disease of amenity turfgrasses in the United States, yet little is known about the source of primary inoculum for this disease. With the exception of a few isolates from the United Kingdom, *Sclerotinia homoeocarpa*, the causal agent of dollar spot, does not produce spores. Consequently, it was assumed that overwintering of this organism in soil, thatch, and plant debris provides primary inoculum for dollar spot epidemics. Overwintering of *S. homoeocarpa* in roots and shoots of symptomatic and asymptomatic creeping bentgrass turfgrass was quantified over the course of a three-year field experiment. Roots did not consistently harbor *S. homoeocarpa*, whereas *S. homoeocarpa* was isolated from 30% of symptomatic shoots and 10% of asymptomatic shoots in the spring of two out of three years. The presence of stroma-like pathogen material on leaf blades was associated with an increase in *S. homoeocarpa* isolation and colony diameter at 48 hpi. Commercial seed has also been hypothesized to be a potential source of initial inoculum for *S. homoeocarpa*. Two or more commercial seed lots of six creeping bentgrass cultivars were tested for contamination with *S. homoeocarpa* using culture-based and molecular detection methods. A viable, pathogenic isolate of *S. homoeocarpa* was isolated from one commercial seed lot and contamination of this lot was confirmed with nested PCR using *S. homoeocarpa* specific primers. A sensitive nested PCR assay detected *S. homoeocarpa* contamination in eight of twelve (75%) commercial seed lots. Seed source, but not cultivar or resistance to dollar spot, influenced contamination by *S. homoeocarpa*. Overall, this research suggests that seeds are a potential source of initial inoculum for dollar spot epidemics and presents the need for further research in this area.

## Introduction

Dollar spot, caused by the fungus *Sclerotinia homoeocarpa*, is the most important disease of turfgrass in North America. It is responsible for the largest disease management expenditures in turfgrass settings throughout the world [1], [2], [3]. Under intense disease pressure, fungicide application costs for dollar spot management may exceed $170/ha per year on high-value golf course greens and fairways [4]. These high costs, coupled with concerns over the environmental impacts of fungicide use and the prevalence of fungicide resistance in *S. homoeocarpa* populations have motivated the development of alternative methods for dollar spot management [3], [5].

The initial descriptions of *S. homoeocarpa* by Bennett [6] reported three strain types for this fungus: Perfect, ascigerous, and nonsporiferous. Since that time, researchers have shown that only *S. homoeocarpa* isolates collected from fescues (*Festuca* sp.) in the United Kingdom are capable of producing conidia or fertile apothecia [6], [7], [8]. Original strains of *S. homoeocarpa* from Australia and the United States examined by Bennett [6] were categorized as nonsporiferous and subsequent studies found that *S. homoeocarpa* isolates from outside the United Kingdom produce apothecial initials but fail to mature and produce sexual ascospores [9], [10]. Consequently, vegetative mycelia and substratal stroma, which is formed by *S. homoeocarpa* under adverse conditions, are believed to be the primary means of survival for this pathogen [1], [11].

The stroma formed by *S. homoeocarpa* consists of a thin, plate-like mat of melanized hyphal cells and lacks the structural features of true sclerotia [10], [12]. With the exception of *S. homoeocarpa*, this type of indeterminate stroma is formed by saprophytic fungi and has been termed a survival structure [12]. Only a single report associates the presence of stroma on leaf blades with overwintering of *S. homoeocarpa* [9]. In that study, the pathogen was isolated from less than 10% of symptomatic leaf blades after a three-month winter incubation period and a considerable number of these isolations were made from leaf blades with visible stroma [9]. While this research provided support for the ability of *S. homoeocarpa* to survive the winter months in dormant turf, it was conducted in artificially constructed environments that may not reflect the true overwintering capacity of *S. homoeocarpa* and was restricted to a single year in a single location [9]. Since *in planta* overwintering is considered an important source of initial inoculum for this pathogen, additional years of data and a more in-depth investigation of *S. homoeocarpa* overwintering are warranted [3].

Dollar spot is ubiquitous in golf course settings and appears within a year of seeding, even following methyl bromide fumigation [14], [15]. The presence of dollar spot after methyl bromide application coupled with evidence that *S. homoeocarpa* grows poorly in soil without plant debris, such as a newly constructed turf green or fairway, suggests that this is not a soil-borne pathogen [16]. Further, lack of spore production by *S. homoeocarpa* isolates originating outside of the United Kingdom indicates that long-distance dispersal via wind or rain is unlikely [9], [10]. Geostatistical analysis of dollar spot epidemics demonstrated limited spread of this pathogen within a season, indicating that equipment does not contribute to spread of inoculum. Consequently, the authors concluded that epidemics initiated from individual clusters of infection centers [13].

Introduction of the pathogen with turfgrass seed is a possible source of long-distance dissemination for *S. homoeocarpa* that corresponds with initiation of epidemics from aggregated areas in a turfgrass sward. In the United States, the vast majority of cool-season turfgrass seed is produced in the Pacific Northwest [17], [18]. There is also limited genetic diversity in *S. homoeocarpa* isolates collected from cool-season grasses in the United States and Canada [19], [20], [21]. Conversely, *S. homoeocarpa* isolates collected from warm-season grasses, which are vegetatively propagated and cultivated in many regions, have much greater genetic diversity than their C3 counterparts [17], [22], [23]. We hypothesize that one explanation for this difference in genetic diversity of C3 and C4 isolates could be dissemination of the pathogen with seed or vegetative propagation material. The finding that seeding rate of both Kentucky bluegrass [24] and fine fescue [14] is positively correlated with dollar spot severity supports the hypothesis that *S. homoeocarpa* may be dispersed with seed. Although this correlative evidence suggests that seed may be a means spreading *S. homoeocarpa*, commercial seed has not previously been evaluated as a possible source of inoculum for *S. homoeocarpa*.

In the present study, we evaluated both *in planta* overwintering of *S. homoeocarpa* and the prevalence of *S. homoeocarpa* contamination in commercial creeping bentgrass seed. The goal of the study was to identify sources of primary inoculum for the dollar spot pathogen in order to advance understanding of dollar spot epidemiology. The specific objectives of this research were to determine: (1) the rate of pathogen overwintering in roots and shoots of symptomatic and asymptomatic creeping bentgrass turf; (2) the importance of stroma in *S. homoeocarpa* overwintering; and (3) the viability, pathogenicity, and prevalence of *S. homoeocarpa* on commercial creeping bentgrass seed.

## Materials and Methods

Field studies were conducted at the O.J. Noer Turfgrass Research Facility. This site is owned by the University of Wisconsin-Madison and is available to all University of Wisconsin-Madison turfgrass research faculty and students. The O.J. Noer Turfgrass Research Facility is located in Verona, WI (43.024181, −89.534294). The turfgrass species sampled, creeping bentgrass, is not considered an endangered or protected species.

### Study site and sample collection

The overwintering study was conducted on a United States Golf Association sand-based creeping bentgrass (*Agrostis stolonifera* L.; cv. ‘Penncross’) putting green maintained at a height of 0.40 cm and located at the University of Wisconsin-Madison's O.J. Noer Turfgrass Research and Education Facility in Verona, Wisconsin (43.024181, −89.534294). The same site was resampled in all three years of the study. In each year, late season dollar spot development permitted sampling from symptomatic turf at the margin of dollar spot infection centers and asymptomatic turf that was not within dollar spot infection centers. Fall sampling was conducted in December of all three years, after the ground had frozen but prior to lasting snow cover. Spring sampling was conducted in late-March to mid-April of each year, after the ground had thawed and snow cover had melted (Table 1). Samples were collected using a standard 2 cm diameter soil probe to a depth of approximately 8 cm to allow for collection of root tissue. In the first winter of the study, 20 samples were collected from both symptomatic and asymptomatic turf. Twenty-five samples were collected in the second and third years of the study. Symptomatic samples were collected from the perimeter of the arbitrarily selected dollar spot infection center. Asymptomatic samples were collected from symptomless turf approximately 15 cm from each infection center; thus, a symptomatic and an asymptomatic sample collected for each infection center. Infection centers were marked with golf tees and were resampled in the spring to allow direct comparisons of fall and spring isolation rates. All samples were collected in individual zip-lock plastic bags and stored at 4°C until plating (<72 hpi).

**Table 1:** Sampling dates for *S. homoeocarpa* overwintering sample collections.

Year	Season	Sampling Date	No. of Samples
2010–2011	Fall	12/1/2010	20
2010–2011	Spring	3/24/2011	20
2011–2012	Fall	12/7/2011	25
2011–2012	Spring	3/29/2012	25
2012–2013	Fall	12/17/2012	25
2012–2013	Spring	4/16/2013	25

### Sample plating and *S. homoeocarpa* isolation

Intact soil cores were surface disinfested prior to plating by submerging in 10% Clorox (0.06% sodium hypochlorite) for 15 s and gently massaging the root area to remove attached sand. Disinfestation was immediately followed by two 30 s rinses in deionized water and samples were then dried on fresh paper towels. Sterile forceps were used to select and plate individual leaf blades and roots onto antibiotic-amended PDA (BD Difco, Sparks, MD; amended with 100 mg/L each tetracycline, chloramphenicol, and streptomycin sulfate). For each sample, four leaf blades and four root pieces were placed on two replicate 50 mm diameter petri dishes (Fisher Scientific, Hanover Park, IL), resulting in a total of eight organ subsamples per sample. All petri dishes were inverted and incubated at ambient temperature (22±2°C) for 48 h.

From 48–96 h after plating, fungal colonies resembling *S. homoeocarpa* were marked and sub-cultured onto fresh antibiotic-amended PDA to obtain pure cultures. In the first year of the study, colony morphology and sequencing of the internal transcribed spacer (ITS) 1 region with the primer pair ITS1/ITS2 were used to positively confirm *S. homoeocarpa* isolations. Fungal DNA was extracted from freshly harvested mycelia of *S. homoeocarpa* using a modified CTAB extraction protocol [25] and fungal ITS region-specific primers ITS1 and ITS2 [26] were used for amplification and sequencing of fungal DNA. DNA sequencing was performed at the University of Wisconsin-Madison DNA Sequencing Facility (Madison, WI). In subsequent years, colony morphology alone was used to confirm *S. homoeocarpa* isolation.

Stroma plating experiments used a procedure similar to that described above. A dissecting microscope was used to visually identify leaf blades with and without stroma-like material from samples previously identified as positive for *S. homoeocarpa* isolation in fall 2012 and spring 2013. Because dollar spot lesions on closely mown creeping bentgrass leaf blades rarely have the distinct reddish brown borders associated with stroma formation in other grasses, dark material embedded in tissue with dollar spot lesions was considered stroma-like. Leaf blades with and without stroma-like material were plated individually in 50 mm diameter petri dishes and *S. homoeocarpa* isolation and identification were repeated as above. Colony diameters were measured at 48 h after plating and were calculated by averaging the lengths of two perpendicular transects across the colony. In total, 20 leaf blades with and 20 leaf blades without stroma-like material were used in these studies. Sample size was constrained by the limited number of leaf blades with stroma-like material.

### Seed sources

Samples from twelve commercial CRB seed lots were obtained directly from seed production companies. CRB cultivars for seed assays were selected based on their resistance to dollar spot in National Turfgrass Evaluation Program trials from 2009–2011 (http://www.ntep.org; Table 2). A minimum of two seed lots per cultivar were tested for *S. homoeocarpa* contamination by both culture-based and molecular detection methods.

**Table 2:** Seed sources, cultivars, and National Turf Evaluation Program performance results for creeping bentgrass commercial seed lots used in *S. homoeocarpa* culture-based and molecular seed detection studies.

Source	Cultivar	NTEP Rating^2^	Resistance Designation
Seed Research of Oregon	96-2	4.6^3^	VS
Mountain View Seed	Shark	5.3	MS
Tee-2-Green	Penncross	5.5	MS
Seed Research of Oregon	SR1150	6.3	R
Lebanon Turf	Declaration	7.4	VR
Seed Research of Oregon	Focus^1^	7.9	VR

1 The experimental name for cv. ‘Focus’ in NTEP trials was ‘GMC comp’.

2 NTEP ratings provided are the average from dollar spot trials in 2009, 2010, and 2010 that included all six cultivars.

3 Ratings are based on turfgrass quality and are given on a 0–9 scale; 9 = highest turf quality/resistance to dollar spot and 0 = lowest turf quality/resistance to dollar spot.

### Culture-based detection

#### Individual seed plating

In an experimental replicate, a total of 1000 seeds from each seed lot were plated on two fungicide-amended media semi-selective for *S. homoeocarpa* [27], one with 5 ppm azoxystrobin and the other with 0.1 ppm triticonazole, in 100 mm diameter petri plates (Fisher Scientific, Hanover Park, IL). Twenty-five seeds were placed on each petri dish, resulting in a total of 20 plates of each medium and 40 plates total per seed lot in each experimental replicate. A dissecting needle was used to select and plate individual seeds. The entire experiment was repeated three times; thus, an overall total of 3,000 seeds per lot were plated. For each experimental replicate, plates were sealed, inverted, and incubated at 22±2°C for 72–120 h. Fungal colonies resembling *S. homoeocarpa* were marked and positively identified as described above for overwintering samples. To allow for identification of other fungal species commonly present in commercial CRB seed, the ten most common fungal colony morphotypes were noted and isolated in pure culture. DNA was extracted, using methods described above [25], from fresh mycelia of these cultures and the ITS 1 region was sequenced with the fungal ITS-region primers ITS1/ITS2 [25].

#### Enrichment and dilution plating

Potato dextrose broth (PDB) was prepared according to the manufacturer's instructions and amended with 100 mg/L of the antibiotics streptomycin sulfate, chloramphenicol, and tetracycline and 25 ppm azoxystrobin (Heritage TL; Syngenta, Greensboro, NC). HCl was added to the medium to decrease the pH to four. Twenty mL of antibiotic and fungicide-amended PDB at pH 4 and 0.25 g of seed from each commercial seed lot were added to 12 sterile flasks. Flasks were sealed with a double layer of parafilm and incubated with constant shaking for 72 h at ambient temperature (22±2°C). After 72 h, the suspension was homogenized in a commercial blender and four 10-fold serial dilutions were made for each sample. Three 0.5 mL aliquots of each dilution were plated onto separate azoxystrobin-amended PDA plates prepared as described above, except that PDA was substituted for PDB. All plates were incubated for 24–48 h. Potential *S. homoeocarpa* colonies were then marked and sub-cultured to obtain pure cultures, as previously described.

Pathogenicity assays with *S. homoeocarpa* isolates collected from seed were conducted as follows. Four-week-old CRB (cv. ‘Penncross’) plants were inoculated by placing two agar plugs, collected from the edge of four day-old fungal cultures, deep within the center of the turfgrass canopy. Mock-inoculated controls were included for comparison. All pots in the experiment were randomized in a plastic flat and covered with a humidity dome to promote infection. Four-week-old barley (cv. ‘Parkland’) plants were inoculated using the parafilm sachet method [28] and control plants were mock-inoculated with PDA plugs. Disease symptoms on both CRB and barley were photographed at 120 hpi. Both CRB and barley plants were cultivated in a growth room at 24±2°C with a 14 h day-length and inoculations were performed under these conditions. Barley was used in these assays because characteristic *S. homoeocarpa* lesions form on this host in controlled environment conditions and are more easily visualized than in creeping bentgrass.

### Molecular detection

For nested and quantitative PCR, primer sets were designed to the outside and inside, respectively, of a previously described primer set specific to the *S. homoeocarpa* EF1α gene [29] (Table 3). The NCBI Primer-BLAST tool (http://www.ncbi.nlm.nih.gov/tools/primer-blast/) was used to design primers specific to the *S. homoeocarpa* EF1α DNA sequence (GenBank Accession DQ448301).

**Table 3:** Primers used for molecular detection of *S. homoeocarpa* in creeping bentgrass commercial seed lots.

Primer Name	Sequence	Prod. Length (bp)	Use	Reference
EF1α_F	CGGTATGACTTCTCCACCTTTC	219	Nested PCR	Al-Elmagid et al. 2013
EF1α_R	GAACCCTTTCCCATCTCCTT		Nested PCR	Al-Elmagid et al. 2013
EF1α_Outer_F	CGGTAAGCAGAACCCTCGAC	554	Primary PCR	This paper^1^
EF1α_Outer_R	CAGCTTGGGAGGTACCAGTG		Primary PCR	This paper
EF1α_Nest_F	TTATCGGGTTGCGTTTTCTC	102	Q-PCR	This paper
EF1α_Nest_R	AACGGGTTAGCAAAGGGATT		Q-PCR	This paper

1 Primers developed in the present research were designed using the NCBI Primer-BLAST tool with the DNA sequence for the *S. homoeocarpa* EF1α input as the sole target sequence. Primer specificity was checked by sequencing of primary and nested PCR products and qPCR melt curve analysis.

Seed DNA was extracted from ten 50 mg subsamples of each seed lot using the Qiagen DNeasy Plant Mini-Kit (Qiagen, Carlsbad, CA) according to the manufacturer's instructions except with slight modifications in sample preparation. Specifically, seeds were ground in Lysing Matrix A tubes (MP Biomedicals, Irvine, CA) containing two 0.64 cm diameter ceramic beads with a FastPrep-24 (MP Biomedicals, Irvine, CA). Samples were homogenized twice for 40 s at 6 m s^−1^ in Qiagen DNeasy kit buffer AP1. RNAse A was added to samples after homogenization but otherwise the manufacturer's protocol was followed. DNA quantity and quality were assessed with an ND-1000 Spectrophotomer (NanoDrop Technologies, Wilmington, DE).

Both primary and nested PCR reactions were performed in 25 µL reaction volumes with GoTaq Colorless PCR Master Mix (Promega, Madison, WI), following the manufacturers protocol. Primary reactions contained: 12.5 µL GoTaq Colorless PCR Master Mix, 2 µL seed DNA, 0.2 µM of primers EF1α_outerF and EF1α_outerR, and nuclease free water (NFW). Nested reaction mixtures were identical, except that 2 µL of primary PCR products were used as template and the EF1α primer set was used. Positive control primary PCR products were diluted 1∶30 with NFW. Cycling conditions for primary and nested PCR included an initial two minute denaturation period at 95°C; 30 cycles of 30 s denaturation at 95°C, 30 s annealing at 56°C and 52°C for primary and nested PCR, respectively, and 30 s elongation at 72°C; and a final five minute elongation period. A positive control of seed DNA spiked with *S. homoeocarpa* genomic DNA and a no-template negative control were included in all runs and PCR was performed in a MasterCycle Pro S (Eppendorf, Hamburg, Germany). Five µL of all primary and nested PCR products were mixed with 1 µL of 6× DNA loading dye and subject to electrophoresis in a 1% agarose gel stained with SYBR Safe DNA Gel Stain (Invitrogen, Carlsbad, CA). Gels were visualized with UV light in a Universal Hood II Gel Doc System (BioRad, Hercules, CA) and analyzed with Quantity One imaging software (BioRad, Hercules, CA). Positive bands were excised from the gel with a sterile razor blade and cleaned up with the Wizard SV Gel and PCR Clean-Up System (Promega, Madison, WI) then sequenced at the University of Wisconsin-Madison DNA Sequencing facility (Madison, WI). NCBI BLASTn was used to confirm sequences were specific to the *S. homoeocarpa* EF1- α gene sequence.

Quantitative PCR (qPCR) reactions were performed in hard-shell 96-well skirted PCR plates and sealed with Microseal ‘B’ adhesive seals (BioRad, Hercules, CA). qPCR reactions contained 1× SsoFast EvaGreen Supermix, 8 µL template (seed DNA or EF1α plasmid DNA standard), 0.2 µM EF1α_nest_F and EF1α_nest_R, and NFW for a final reaction volume of 20 µL. A standard curve with concentrations of EF1α plasmid DNA ranging from 10^6^ to one copy/µL was included on each plate and all DNA samples were assayed in triplicate. Cycling conditions included an initial 2 minute denaturation at 98°C, followed by 40 cycles of 98°C for 2 s and annealing at 55°C for 2 s. A melt curve was created after the final cycle by increasing the temperature from 70–90°C in 0.2°C intervals and holding at each temperature for 10 s. qPCR reactions were performed in a CFX-96 Real-Time System (Bio-Rad, Hercules, CA). The plasmid used for generation of standard curves and copy number estimation was constructed by cloning *S. homoeocarpa* EF1α sequence amplified by our primers into a pGEM-T Easy plasmid (Promega, Madison, WI) according to the manufacturer's instructions. *Escherichia coli* DH5α was transformed with plasmid containing the target sequence and plasmid DNA was purified from overnight cultures of *E. coli* containing the cloned target using the Wizard Plus SV Miniprep DNA purification system (Promega, Madison, WI), as instructed by the manufacturer. A NanoDrop was used to quantify purified plasmid and copy number was calculated according to the method of Frost and colleagues [30].

### Statistical analysis

Statistical analyses were performed using the generalized linear mixed model procedure (PROC GLIMMIX) in SAS version 9.3 [31]. Studentized residuals and tests in PROC UNIVARIATE were used to assess normality and determine the best fitting distribution for each data set. Full models with all random effects and their interactions with fixed effects were then fit to the appropriate distribution and model fitting criteria (AIC of -2RLL, depending on distribution) were used to select the best fitting model.

Overwintering data was coded as a binary variable (0 = no *S. homoeocarpa* detected; 1 = *S. homoeocarpa* detected) and subject to analysis of variance using the binary distribution. Sample (block) was considered a random effect and all other factors (treatment, organ, and season) were fixed. The full data set was analyzed to test for a season effect. Individual years were then analyzed independently and factors (season or treatment) were sliced to compare specific levels of each factor. Stroma isolation data were also subject to analysis of variance under the binary distribution. Stroma colony diameter data were analyzed using the normal distribution. In both cases, a simple one-way ANOVA was used to test for the main effect of the presence or absence of stroma.

Nested PCR seed detection data were subject to analysis of variance under a normal distribution. Because lots were exclusive to the cultivar from which they came and cultivars were exclusive to the source from which they came, both of these factors were treated as nested in statistical analyses. Source and cultivar (source) were considered fixed effects while lot (cultivar) was treated as a random effect. Fixed treatment effects were assessed with a simple two-way ANOVA and a single degree of freedom orthogonal contrast was used to test for a difference between resistant and susceptible cultivars.

## Results

### Pathogen overwintering

**Figure 1:**
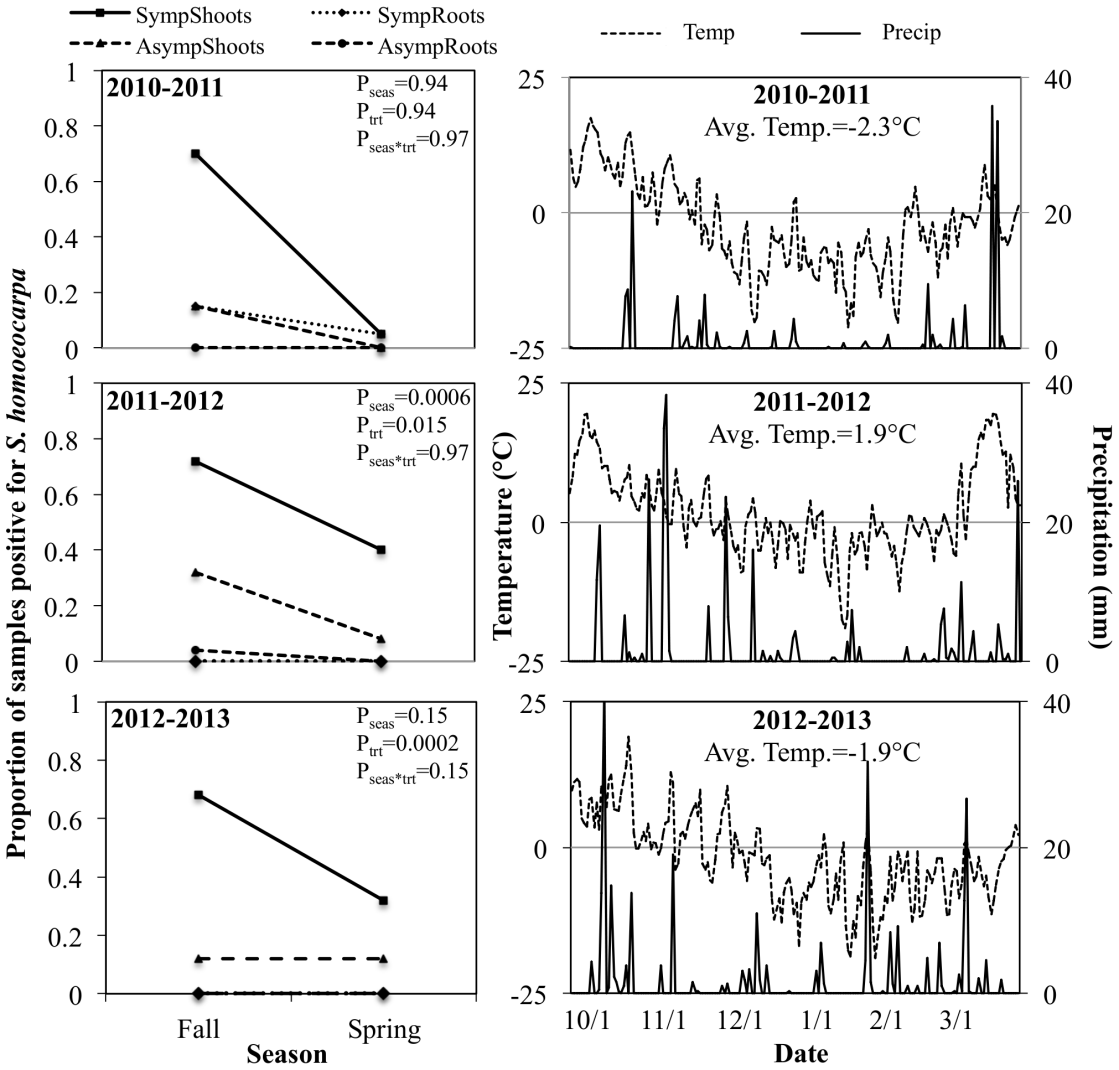
*Sclerotinia homoeocarpa* isolation and weather data for pathogen overwintering studies. Roots and shoots were collected from symptomatic and asymptomatic turf in the fall of each year to determine starting *S. homoeocarpa* populations. The same areas were resampled in the spring to assess pathogen survival. ANOVA values for season, treatment, and season by treatment effects are reported for each year. Weather data includes daily air temperature (dashed line) and precipitation (solid line) averages. The average temperature in 2011–2012 was c. 4°C higher than in the other two years and a major snow events occurred in early November 2011–2012 and early February of 2012–2013 (arrows).

In all three years of this study, the pathogen was isolated from 70% of symptomatic shoots (Fig. 1). *S. homoeocarpa* was isolated from asymptomatic shoots in the fall of all years, but the percentage of positive samples varied, with 15%, 32%, and 12% of samples positive for *S. homoeocarpa* in 2010, 2011, and 2012, respectively (Fig. 1). The percentage of root samples positive for *S. homoeocarpa* was 15% in fall 2010 but negligible in all other seasons. Consequently, roots were not included in statistical analyses of *S. homoeocarpa* overwintering. In the first year of the study, identification by colony morphology was confirmed by sequencing results (File S1); consequently, colony morphology alone was used to identify *S. homoeocarpa* isolates in future years of the study.

The majority of spring isolations of *S. homoeocarpa* were made from samples taken from areas with dollar spot symptoms the previous fall (Fig. 1). The rate of *S. homoeocarpa* spring isolation from symptomatic tissue ranged from 0.05% in spring of 2010 to 40% in spring of 2011. In two of three years of this study, spring isolations of *S. homoeocarpa* were also made from tissue collected from asymptomatic areas in the previous fall (Fig. 1). *S. homoeocarpa* was isolated from 8 and 12% of asymptomatic samples in the spring of 2012 and 2013, respectively. In the 2012–2013 season, there was no difference in the percentage of samples positive for *S. homoeocarpa* in the fall and spring. A single spring isolation of *S. homoeocarpa* was made from roots in spring 2011. *S. homoeocarpa* was not recovered from roots at any other spring sampling date.

Across all years, season had an effect on *S. homoeocarpa* isolation from both symptomatic (*P<0.001*) and asymptomatic (*P = 0.03*) samples. In individual years, a significant effect of season across sample types (symptomatic or asymptomatic) was detected only in Year 2 (Fig. 1). By separating sample types, an effect of season was detected in symptomatic samples for all three years (*P<0.05*) and for asymptomatic samples in Year 2 (*P = 0.03*). Sample type had an effect on *S. homoeocarpa* isolation in the fall of each year (*P<0.05*), but had no effect on spring isolations (*P>0.1*) in any year. No season by sample type interaction was detected in any year of the study (*P>0.1*).

### Stroma

**Figure 2:**
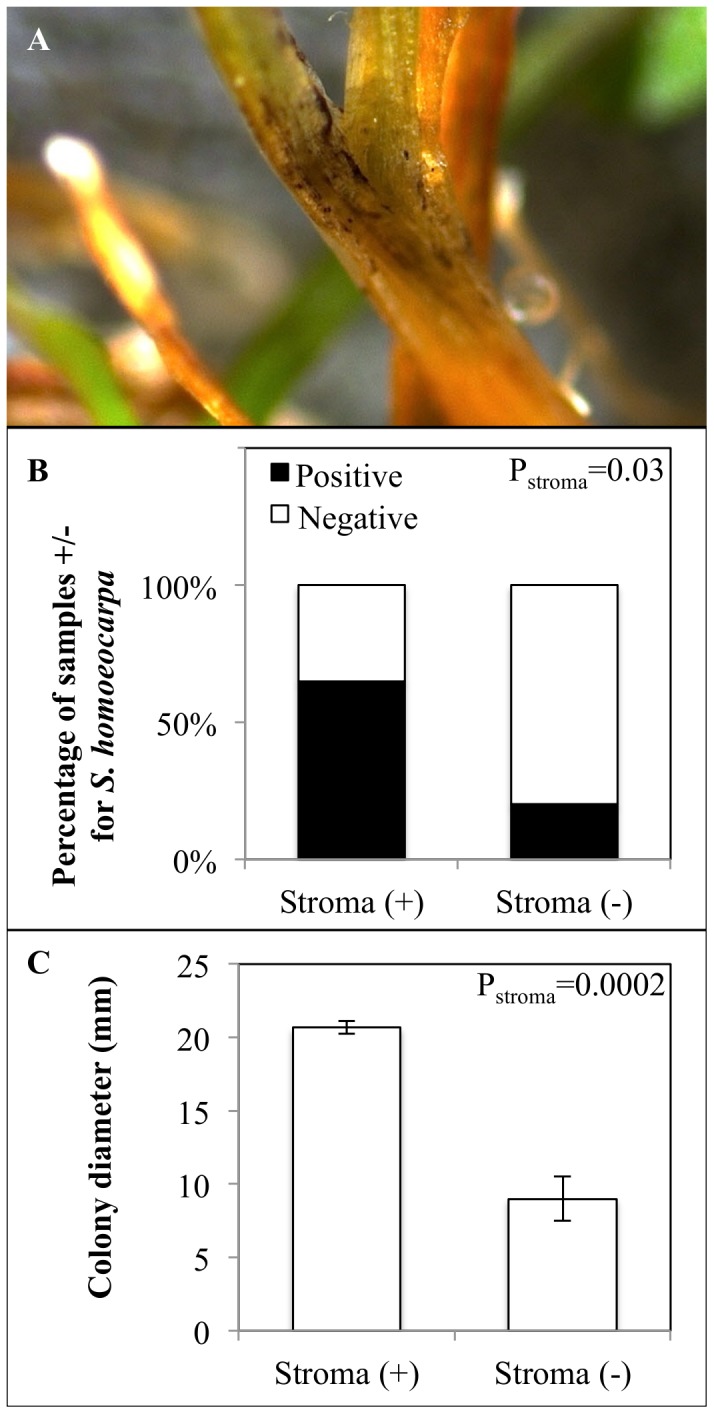
Stroma on creeping bentgrass leaf blades and its effect on isolation on *S. homoeocarpa*. **A**, typical stroma-like material observed on a turfgrass leaf blade was brown in color and appeared as flaky, plate-like material. **B**, The percentage of CRB leaf blades with and without visible stroma from which *S. homoeocarpa* was isolated. P-value from ANOVA using the binary distribution for presence/absence of *S. homoeocarpa* and α = 0.05. **C**, Average diameter of *S. homoeocarpa* colonies 48 h after plating leaf blades with or without visible stroma. P-value is from ANOVA with the normal distribution and α = 0.05. Error bars represent ± one standard error of the mean (n = 5).

Leaf blades with black to brown stroma-like material on or within epidermal tissue were readily identified with a dissecting microscope and were selected as potentially harboring *S. homoeocarpa* stroma (Fig. 2A). Leaf blades serving as non-stroma controls were green to yellow in color and did not appear to contain fungal material. *S. homoeocarpa* was isolated from leaf blades with and without stroma, but the presence of stroma-like material significantly increased both the rate of isolation (*P = 0.03*; Fig. 2B) and growth rate (*P = 0.0002*; Fig. 2C) of *S. homoeocarpa*.

### Culture-based detection

#### Individual seed plating

A single isolate of *S. homoeocarpa* was collected from commercial seed with the culture-based detection method (Table 4). The isolate was collected from Lot 1 of cv. ‘Shark’ on the triticonazole–amended semi-selective medium [27]. Colony morphology (Fig. S1A) and amplification of the ITS1 region by universal primers ITS1/ITS2 [26] (File S1) confirmed the identification of this isolate. Tests on CRB and barley demonstrated pathogenicity and resulting symptoms were similar to those observed with a virulent isolate of *S. homoeocarpa* (Fig. S1B–C). Specifically, symptoms on CRB included water-soaking around the site of inoculation, accompanied with reddish brown lesions on individual leaf blades. Light-tan lesions with reddish brown borders, characteristic of *S. homoeocarpa* infection, developed on barley.

**Table 4:** Results for *S. homoeocarpa* contamination of creeping bentgrass commercial seed lots by culture-based and molecular detection methods.

Cultivar	Lot^1^	Culture-based Detection^2^	Nested PCR^3^ (Subsamples +)	Q-PCR^4^ (Subsamples +)
96-2	1	−	+ (1)	n/a
	2	−	− (0)	n/a
Shark	1	+	+ (5)	− (0)
	2	−	+ (3)	− (0)
Penncross	1	−	+ (1)	− (0)
	2	−	+ (2)	n/a
SR1150	1	−	− (0)	− (0)
	2	−	− (0)	n/a
Declaration	1	−	+ (2)	n/a
	2	−	− (0)	n/a
Focus	1	−	+ (1)	− (0)
	2	−	+ (1)	n/a

1 Two representative lots of each cultivar were selected by for detection of *S. homoeocarpa* contamination by each method; additional lots of ‘Shark’ and ‘Penncross’ seed were tested by the nested PCR detection method only.

2 Three 1,000 seed replicates for all lots were performed by plating 500 seeds from each lot onto two different media semi-selective for *S. homoecarpa* (1,000 seeds total) and repeating three times (3,000 seeds/lot total).

3 Nested PCR was repeated twice for each seed lot; numbers shown in parentheses are the number of subsamples testing positive for *S. homoeocarpa* contamination in each run.

4 Q-PCR detection was ceased after it was determined that PCR inhibitors in seed DNA samples rendered the assay ineffective.

Overall, 16 different fungal colony morphotypes were commonly isolated during seed plating. The most commonly isolated seed contaminant was a pink yeast in the genus *Rhodotorula*. The fungus *Epicoccum nigrum* was the second most commonly isolated seed contaminant. Other frequently isolated fungi included *Trichoderma* sp., *Penicillium polonicum*, *Mucor fragilis*, and *Aureobasidium proteae*. Lack of morphological characteristics needed for identification, inability to extract quality DNA, or poor sequencing results made it difficult to identify many of the fungi isolated.

#### Enrichment and dilution plating

After 24 h of incubation, plates from the enrichment experiment were overrun with Zygomycete fungi; consequently, no *S. homoeocarpa* colonies were identified by this method.

### Molecular detection

#### Nested PCR

Nested PCR using the primary primer set EF1α_Outer and nested primer set EF1α was sensitive enough to detect a single *S. homoeocarpa*-infested seed in 50 mg of seed (Fig. S2). A minimum of two seed lots per CRB cultivar were tested for *S. homoeocarpa* contamination with nested PCR. Eight of the twelve seed lots tested were positive for *S. homoeocarpa* DNA (Table 4), including both seed lots from cultivars ‘Penncross’ and ‘Shark’. The highest contamination was present in ‘Shark’ Lot 1, from which *S. homoeocarpa* was also isolated using the culture-based detection method. Three additional ‘Shark’ seed lots were subsequently tested for *S. homoeocarpa* contamination and two were positive (Table 4). A subset of positive nested PCR products were sequenced (File S1) and their identity confirmed through sequence similarity to the *S. homoeocarpa* EF1α sequence (GenBank Accession DQ448301).

**Figure 3:**
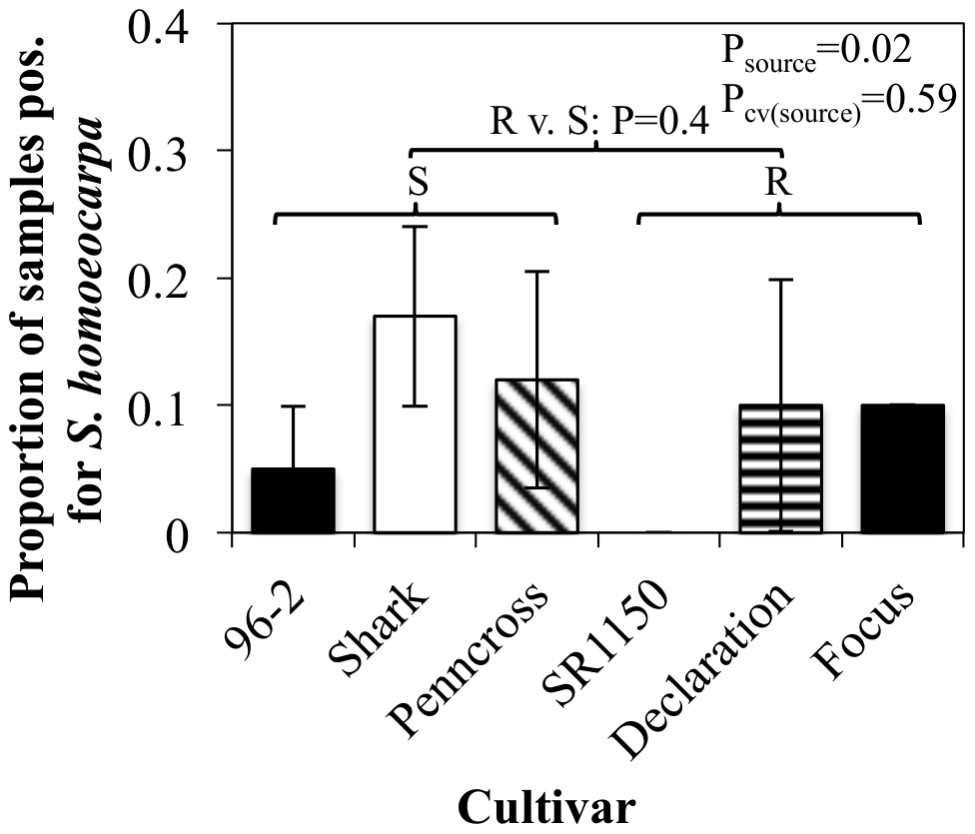
Results of nested PCR detection of *Sclerotinia homoeocarpa* DNA in creeping bentgrass commercial seed lots. Columns reflect the average proportion of samples positive for *S. homoeocarpa* contamination across the two lots of each cultivar. Shading indicates cultivar source: ‘96-2’, ‘Focus,’ and ‘SR1150’ were from a single source while ‘Shark,’ ‘Penncross,’ and ‘Declaration’ were from different seed distributors. Error bars represent ± one standard error of the mean. Source and cv(source) P-values are from ANOVA of the data with the normal distribution and α = 0.05. The P-value for R versus S cultivars was obtained from a pre-planned orthogonal contrast.

Seed source affected detection of *S. homoeocarpa* (*P = 0.01*) based on ANOVA of nested PCR data. CRB cultivar did not affect detection (*P>0.05*). Cultivars were nested within seed source; thus, source by cultivar interaction was not tested. An orthogonal contrast between cultivars with high and low dollar spot resistance, according to NTEP creeping bentgrass variety trials, indicated that dollar spot resistance did not influence detection levels for *S. homoeocarpa* (*P>0.05*; Fig. 3).

#### Quantitative PCR

The primer set EF1α_Nest_F/EF1α_Nest_R had >99% efficiency in standard curves constructed from plasmid DNA and a detection limit as low as 10 target copies/µL template (Fig. S3A). Six seed lots positive for *S. homoeocarpa* by nested PCR were also tested by qPCR. Five of the six lots, including Shark Lot 1, tested negative for *S. homoeocarpa* contamination by qPCR (Table 4). The low success rate of qPCR suggested the presence of PCR inhibitors in seed DNA samples. Decreased detection threshold values following sample dilution and increased threshold values in spiked seed DNA samples over standards with the same target concentration confirmed the presence of PCR inhibitors in seed DNA (Fig. S3B).

## Discussion

This is the first multi-year study on *in planta* overwintering of *S. homoeocarpa*. Overall, our results indicate that *S. homoeocarpa* overwinters moderately well in infected plant material. Previously, the only study on *S. homoeocarpa* overwintering was conducted on Kentucky bluegrass (*Poa pratensis*) at the University of Rhode Island in the winter of 1973–1974 [9]. In that study, *S. homoeocarpa* was recovered from less than 10% of symptomatic leaf blades and never from asymptomatic leaf blades [9]. This research differs significantly from this previous experiment because a different turf species (creeping bentgrass as opposed to Kentucky bluegrass) and a much harsher winter climate were evaluated. Most importantly, this research evaluated *S. homoeocarpa* overwintering under entirely natural conditions, without any manipulation of the host or pathogen until the time of sample collection. In the present research, spring recovery of *S. homoeocarpa* ranged from 5–40% and 0–12% in symptomatic and asymptomatic samples, respectively. The authors of the previous study [9] noted the presence of numerous saprophytic fungi in their samples and degradation of leaf blades by fungal contaminants. While saprophytes were frequently recovered in our study, they did not appear to degrade host tissue in the course of our study. This may be one reason for the higher rates of *S. homoeocarpa* isolation in the present research. It is also plausible that the higher rates of overwintering in our study are a reflection of the methods used—undisturbed dormant infection centers in our experiment, symptomatic leaf blades placed inside plastic straws in the previous experiment.

The rate of fall isolation from symptomatic shoots was similar in all three years of our study, but spring isolation rates varied, indicating that environmental factors may influence winter survival of *S. homoeocarpa*. Consequently, average daily temperature and precipitation data were collected and compared for the three years of our study (Fig. 1B,D,F). *S. homoeocarpa* survival rates were highest in the second year of the study, during which average winter temperatures were approximately 4°C higher than in years one and three. The warmer winter temperatures in 2011–2012 may have promoted overwintering of *S. homoeocarpa* relative to the other two years of this study. Warmer winter temperatures are also associated with increased survival of the turfgrass stem rust pathogen, *Puccinia graminis* subsp. *graminis* on tall fescue and perennial ryegrass [32]. Spring isolations of *S. homoeocarpa* were higher in the third year of our study than in the first year, despite similar average winter temperatures in both years. A heavy snowfall event in late January of 2013 may have insulated *S. homoeocarpa* from fluctuating air temperatures, resulting in a greater number of spring isolations in year three. Direct comparisons of these studies to prior research are difficult because most research on fungal pathogen overwintering focuses on survival of spores and often involves artificial manipulations of the pathogen itself or of infected host material [33], [34], [35]. However, a few studies have shown that fungal pathogens survive harsh winter conditions in host debris that then serves as primary inoculum in the following spring [36], [37], [38].

Increased isolation of *S. homoeocarpa* from leaf blades with stroma in our study agrees with the findings of Fenstermacher [9] on Kentucky bluegrass and supports the general conclusion that stroma is a survival structure for this pathogen [3], [6], [11]. Stroma was not observed on creeping bentgrass roots and isolations of *S. homoeocarpa* from roots were limited, indicating that overwintering in roots does not contribute to primary inoculum for dollar spot epidemics. To our knowledge, this is the first report that stroma aids overwintering of *S. homoeocarpa* in creeping bentgrass. The atypical nature of *S. homoeocarpa* stroma in comparison with other pathogenic fungi in the Sclerotiniaceae family, including the fact that this survival structure is generally restricted to saprophytic fungi, warrant further investigation of the role of this structure in *S. homoeocarpa* biology and epidemiology [12]. Potentially, control methods aimed at decreasing the formation of stroma in the fall could decrease overwintering of *S. homoeocarpa* and reduce initial inoculum for dollar spot epidemics.

The isolation of *S. homoeocarpa* from asymptomatic samples in the spring of 2011–2012 and 2012–2013 was unexpected. Since diseased tissue from the previous year's epidemic was believed to be the source of inoculum for subsequent years [3], [11], [39], detection of this pathogen in asymptomatic material indicates that latent infections may be an important source of overwintering and inoculum. Isolation of *S. homoeocarpa* from asymptomatic turfgrass also may help explain the detection of *S. homoeocarpa* in commercial creeping bentgrass seed. In the Pacific Northwest, where the majority of cool-season turfgrass seed is produced, environmental conditions are generally not suitable for dollar spot epidemics [40]. Asymptomatic infection with *S. homoeocarpa* may allow contamination of seed fields to go unnoticed; thus, leading to harvesting and distribution of seed that appears to be pathogen-free yet is latently contaminated with *S. homoeocarpa*. Symptomless infection arising from seeds has previously been described for *Botrytis cinerea* in lettuce [41] and for many bacterial pathogens. The frequency of asymptomatic colonization of turfgrasses by *S. homoeocarpa* should be investigated further.

To our knowledge, this is the first report of a viable, pathogenic isolate of *S. homoeocarpa* obtained from commercial seed of any turfgrass species. This finding provides evidence that commercial seed may be an important source of inoculum for dollar spot epidemics. A single isolation from the 36,000 total seeds plated in this study may seem trivial, but a kilogram of CRB seed contains two to four million individual seeds [42]. This translates to approximately 70 *S. homoeocarpa*-infected seeds per kilogram of creeping bentgrass seed or a minimum of thousands of infected seeds on a freshly seeded golf course putting green at a standard seeding rates [42]. In the present study, we only detected the presence of *S. homoeocarpa* in commercial seed and did not directly relate this to establishment of dollar spot epidemics in the field. Studies investigating the influence of seed contamination with *S. homoeocarpa* on the development of dollar spot epidemics are a logical and necessary next step.

Eight of twelve (75%) commercial seed lots in our study tested positive for *S. homoeocarpa* with nested PCR. In these lots, the number of positive subsamples ranged from 1–5. The discrepancy between *S. homoeocarpa* detection by the culture-based and molecular methods could be a result of amplification of non-viable pathogen DNA with nested PCR or of contaminant fungi outcompeting *S. homoeocarpa* with the culture-based method. With the nested PCR method, a single *S. homoeocarpa* infested seed in a 50 mg sample, approximately 1 in 450 creeping seeds (0.002% infestation), was detected. This detection rate is higher than the 0.01–0.1% detection rates reported for other molecular detection methods [43], [44], [45] though not as high as that of some qPCR detection methods [46], [47]. We found that qPCR was less sensitive than nested PCR for our samples because turfgrass seed has many associated PCR inhibitors. Though qPCR detection methods are currently popular, regular and nested PCR-based detection methods are still highly effective and provide an efficient means of qualitative pathogen detection [44], [48], [49], [50]. In the future, the nested PCR method could lead to development of a loop-mediated isothermal amplification (LAMP) assay for detection of *S. homoeocarpa* in seed. LAMP assays are less sensitive to PCR inhibitors than conventional and real-time PCR methods and can be performed in the field with minimal equipment or training [51], [52], [53], [54].

Analysis of nested PCR data indicated that seed source but not cultivar resistance to dollar spot had an effect on contamination with *S. homoeocarpa*. This was somewhat surprising as an influence of host cultivar resistance on seed contamination has previously been demonstrated for other cereal pathogens [55], [56]. However, the sample size of two lots from each of six cultivars limited our power to detect significant differences between cultivars. In the future, it will be necessary to screen more seed lots for *S. homoeocarpa* contamination. A number of factors could explain the effect of source on seed lot contamination. Previous field history or environmental conditions may differ between production fields used by individual seed companies. Climatic conditions in the Pacific northwest, where the majority of creeping bentgrass seed is grown, are fairly stable and unlikely to vary drastically from one company's fields to the next [17]. It is possible, however, that certain fields or areas within a field have microclimates conducive to plant infection by *S. homoeocarpa*. Alternatively, management or harvest practices may vary between companies and contribute to the differences observed. It is unclear whether *S. homoeocarpa* is harbored on or in the seed itself or associated with debris mixed in with the seed. If the latter is the case, seed with minimal debris may also have lower rates of *S. homoeocarpa* contamination. Future experiments to determine the source of *S. homoeocarpa* in commercial seed will help to identify practices that promote or limit contamination of seed with this pathogen.

## Supporting Information

Figure S1 ***Sclerotinia homoeocarpa*** **isolate ‘Shark’ obtained from Shark Lot 1 using culture-based detection of semi-selective medium.** **A**, Colony morphology of the isolate ‘Shark’ obtained from CRB cv. ‘Shark’ commercial seed lot 1. **B**, Symptoms produced by mock-inoculated control, virulent *S. homoeocarpa*, and seed *S. homoeocarpa* isolate ‘Shark’ on creeping bentgrass (cv. ‘Penncross’) at 5 dpi. Similar symptoms were produced for each treatment in six biological replicates. **C**, Symptoms produced by mock-inoculated control, virulent *S. homoeocarpa*, and seed *S. homoeocarpa* isolate ‘Shark’ on barley (cv. ‘Parkland’) at 5 dpi. Similar symptoms were produced for each treatment in six biological replicates.
(TIF)

Figure S2 **Sensitivity of** ***Sclerotinia homoeocarpa*** **-specific primers in primary and nested PCR.** Primary PCR was run with *S. homoeocarpa* specific primers EF1α_OuterF/EF1α_OuterR, with an expected product size of 554 bp, and contained 2 µL of seed DNA from seed samples spiked with the indicated number of artificially-infested CRB seeds prior to DNA extraction. Nested PCR was performed using *S. homoeocarpa* specific primers EF1α_F/EF1α_R, with a product size of 219 bp internal to EF1α_OuterF/EF1α_OuterR, and contained 2 µL of primary PCR products diluted 1∶30 in nuclease free water. The negative control was treated exactly as samples except that 2 µL of NFW replaced template DNA in the primary PCR. Five µL of sample were mixed with 1 µL of DNA loading dye and run in a 1% agarose gel in TBE buffer along with 5 µL of TrackIt 100 bp DNA ladder (Invitrogen, Carlsbad, CA).
(TIF)

Figure S3 **Q-PCR for molecular detection of** ***Sclerotinia homoeocarpa*** **DNA in creeping bentgrass commercial seed lots.** **A**, Standard curve of EF1α plasmid DNA with the primer set EF1α_NestF/EF1α_NestR indicating near 100% primer efficiency. **B**, Q-PCR run with various controls to reveal the presence of PCR inhibitors: Spike, Spike^−1^, Spike^−2^—Ten-fold dilution series of seed DNA mixed with 1/10 volume EF1α plasmid DNA at a starting concentration 1×10^6^.; Check, Check^−1^, Check^−2^—Ten-fold dilution series of a seed DNA sample positive by nested PCR but negative by Q-PCR. Decreasing Cq value with dilution indicates presence of inhibitors in the original DNA sample.
(TIF)

File S1 **DNA sequences from overwintering** ***Sclerotinia homoeocarpa*** **isolates, isolate ‘Shark’ obtained from commercial seed, and** ***S. homoeocarpa*** **EF1α sequences from seed lots positive by nested PCR.**
(DOCX)
