# Supplementary figures and images for: Sclerotinia homoeocarpa Overwinters in Turfgrass and Is Present in Commercial Seed
Source: PLoS One. 2014 Oct 21;9(10):e110897. doi: 10.1371/journal.pone.0110897 (PMC4204931; doi:10.1371/journal.pone.0110897)

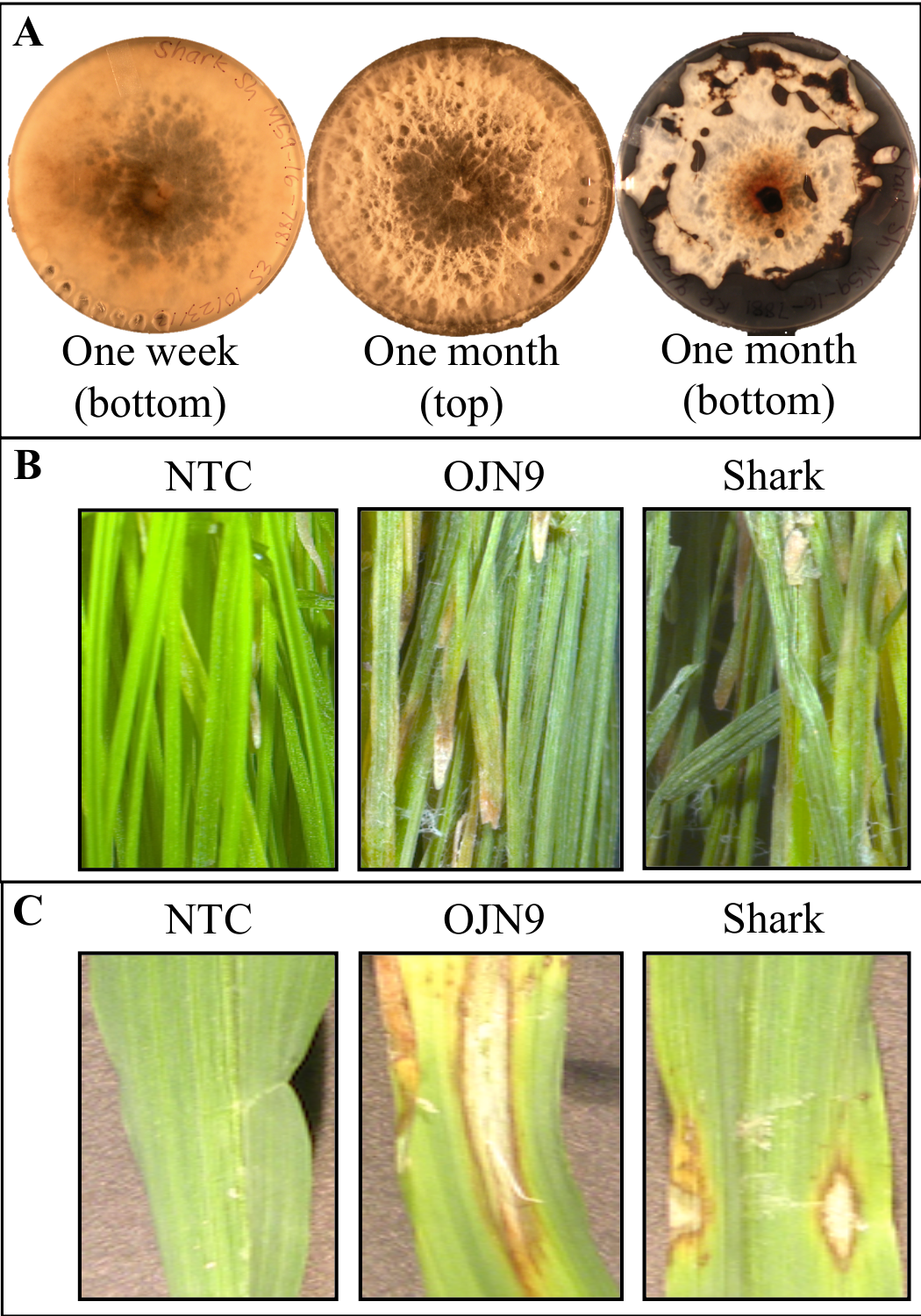

Supplement: Figure S1 — Sclerotinia homoeocarpa isolate ‘Shark’ obtained from Shark Lot 1 using culture-based detection of semi-selective medium. A, Colony morphology of the isolate ‘Shark’ obtained from CRB cv. ‘Shark’ commercial seed lot 1. B, Symptoms produced by mock-inoculated control, virulent S. homoeocarpa, and seed S. homoeocarpa isolate ‘Shark’ on creeping bentgrass (cv. ‘Penncross’) at 5 dpi. Similar symptoms were produced for each treatment in six biological replicates. C, Symptoms produced by mock-inoculated control, virulent S. homoeocarpa, and seed S. homoeocarpa isolate ‘Shark’ on barley (cv. ‘Parkland’) at 5 dpi. Similar symptoms were produced for each treatment in six biological replicates. (TIF) [file pone.0110897.s001.tif]

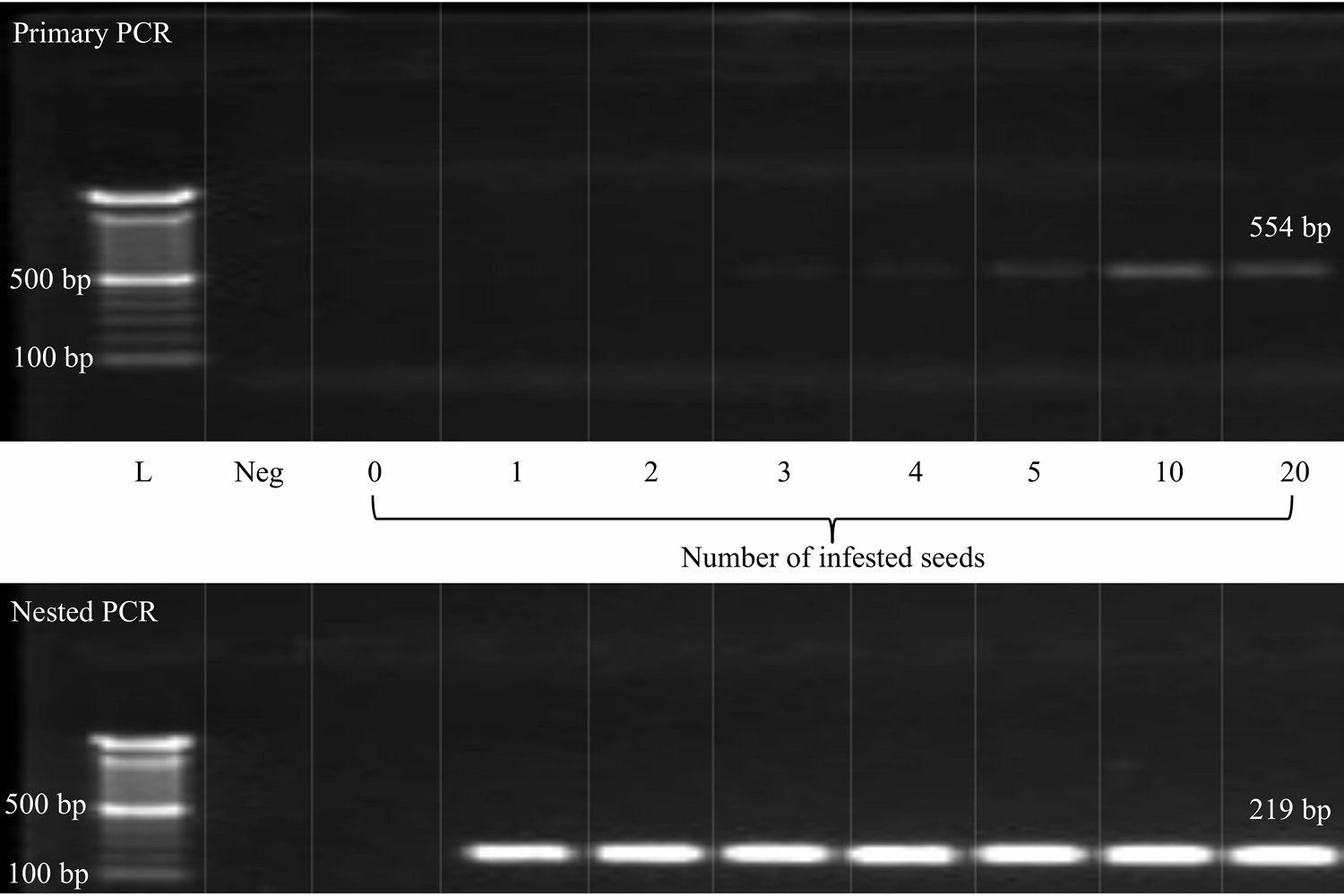

Supplement: Figure S2 — Sensitivity of Sclerotinia homoeocarpa -specific primers in primary and nested PCR. Primary PCR was run with S. homoeocarpa specific primers EF1α_OuterF/EF1α_OuterR, with an expected product size of 554 bp, and contained 2 µL of seed DNA from seed samples spiked with the indicated number of artificially-infested CRB seeds prior to DNA extraction. Nested PCR was performed using S. homoeocarpa specific primers EF1α_F/EF1α_R, with a product size of 219 bp internal to EF1α_OuterF/EF1α_OuterR, and contained 2 µL of primary PCR products diluted 1∶30 in nuclease free water. The negative control was treated exactly as samples except that 2 µL of NFW replaced template DNA in the primary PCR. Five µL of sample were mixed with 1 µL of DNA loading dye and run in a 1% agarose gel in TBE buffer along with 5 µL of TrackIt 100 bp DNA ladder (Invitrogen, Carlsbad, CA). (TIF) [file pone.0110897.s002.tif]

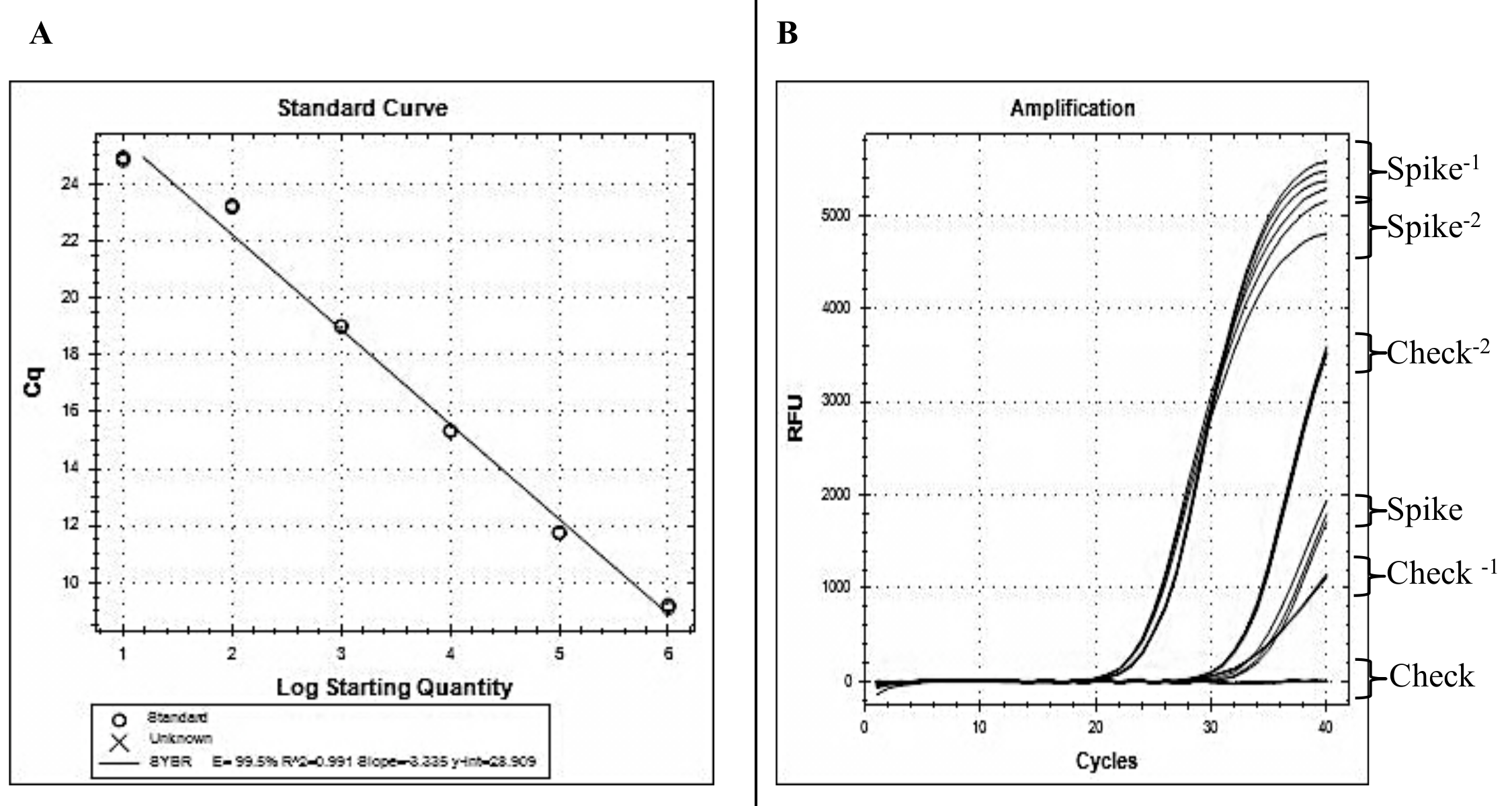

Supplement: Figure S3 — Q-PCR for molecular detection of Sclerotinia homoeocarpa DNA in creeping bentgrass commercial seed lots. A, Standard curve of EF1α plasmid DNA with the primer set EF1α_NestF/EF1α_NestR indicating near 100% primer efficiency. B, Q-PCR run with various controls to reveal the presence of PCR inhibitors: Spike, Spike−1, Spike−2—Ten-fold dilution series of seed DNA mixed with 1/10 volume EF1α plasmid DNA at a starting concentration 1×10^6^.; Check, Check−1, Check−2—Ten-fold dilution series of a seed DNA sample positive by nested PCR but negative by Q-PCR. Decreasing Cq value with dilution indicates presence of inhibitors in the original DNA sample. (TIF) [file pone.0110897.s003.tif]
