# Supplementary material for: Sclerotinia homoeocarpa Overwinters in Turfgrass and Is Present in Commercial Seed
Source: PLoS One. 2014 Oct 21;9(10):e110897. doi: 10.1371/journal.pone.0110897 (PMC4204931; doi:10.1371/journal.pone.0110897)
Supplement: File S1 — DNA sequences from overwintering Sclerotinia homoeocarpa isolates, isolate ‘Shark’ obtained from commercial seed, and S. homoeocarpa EF1α sequences from seed lots positive by nested PCR. (DOCX) [file pone.0110897.s004.docx]

**PCR confirmation of *Sclerotinia homoeocarpa* isolates from 2010-2011 overwintering**

>Sclerotinia homoeocarpa isolate 01 from OJN creeping bentgrass fall 2010; primers ITS/ITS2

CCATCCCCCGGAAATCCAAGGGGCGCAATGTGCGCTTCAAAGATTCGATGATTCACGTGAATTCTGCAATTCACATTACTTATCGCATTTCGCGTGCGATTCTTCATCGATGCCAGAACCAAGAGATCCGTTGTTGAAAGTTTGAACTATTAGNNNGTCACNCAGACGACACTGACAATNCANANTNGTGATTTCCNNCCGGAA

> Sclerotinia homoeocarpa isolate 02 from OJN creeping bentgrass fall 2010; primers ITS/ITS2

GCCCCCCGGAAATCCAAGGGGGCGCAATGTGACGCTTCAAAGGATTCGATGATTCACGTGAATTCGTGCAATTCACATTACATTATCGCATTTCGCGTGCGATTCTTCATCGATGCCAGAACCAAGAGATCCGTTGTTGAAAGTTTGAACTATTAGNNNGNCACNCAGACGACACTGACAATNCANANTNGTGATTTCCNCCCGGA

> Sclerotinia homoeocarpa isolate 03 from OJN creeping bentgrass fall 2010; primers ITS/ITS2

GATTTCNNGCNTTGNNNNNTCNACNTCCCCCGGAAATCCAAGGGGCGCAATGTGCGCTTCAAAGATTCGATGATTCACTGAATTCTGCAATTCACATTACTTATCGCATTTCGCGTGCGATTCTTCATCGATGCCAGAACCAAGAGATCCGTTGTTGAAAGTTTGAACTATTAGNNNGNCACNCAGACGACACTGACAATNCANANTNGTGATTTCCNCCCGGA

> Sclerotinia homoeocarpa isolate 04 from OJN creeping bentgrass fall 2010; primers ITS/ITS2

NANTTNNNGGGANGNNNNNNNCNANANCCCCCCGGGAAATCCAAGGGGTCGCAATGTGCGCTTCAAAGGATTCGGATGATTCACGTGAAATTCGTGCAATTCACATTACCTTATCGCATTTCGCGTGCGATTCTTCATCGATGCCAGAACCAAGAGATCCGTTGTTGAAAGTTTTAACTATTAGATNGTCACTCAGACGACACTGACAATTCANANTTGTGATTTNCCNCCGGA

> Sclerotinia homoeocarpa isolate 05 from OJN creeping bentgrass fall 2010; primers ITS/ITS2

GNTTTTNNGCNNGNNNNNNNNCCGNCCCCCGGAATCCAAGGGGCGCAATGTGCGCTTCAAAGATTCGATGATTCACGTGAATTCTGCAATTCACATTACTTATCGCATTTCGCGTGCGATTCTTCATCGATGCCAGAACCAAGAGATCCGTTGTTGAAAGTTTTAACTATTAGANNGTCACTCAGACGACACTGACAATTCANANTTGTGATTTNCCNCCGGA

>Sclerotinia homoeocarpa isolate 06 from OJN creeping bentgrass fall 2010; primers ITS/ITS2

GNTNTCCGGGGGAGNNNNNNNNNCCANCCCCCCGGAATCCAAGGGGCGCAATGGTGCGCTTCAAAGATTCGATGATTCACGTGAATTCGTGCAATTCACATTACTTATCGCATTTCGCGTGCGATTCTTCATCGATGCCAGAACCAAGAGATCCGTTGTTGAAAGTTTTAACTATTAGNNNGTCACNCAGACGACACTGACAATNCANANTTGTGATTTNCNNCCGGA

> Sclerotinia homoeocarpa isolate 07 from OJN creeping bentgrass fall 2010; primers ITS/ITS2

GATTTCCCGGCTTGNNNNNNNNCNNCCCCCGGAATCCAAGGGGCGCAATGTGCGTTCAAAGATTCGATGATTCACTGAATTCTGCAATTCACATTACTTATCGCATTTCGCGTGCGATTCTTCATCGATGCCAGAACCAAGAGATCCGTTGNTGAAAGTTTGAACTATTAGNGNGNCACNCAGACGACACTGACAATNCANANTTGTGNTTTNCNCCCGGAA

> Sclerotinia homoeocarpa isolate 08 from OJN creeping bentgrass fall 2010; primers ITS/ITS2

CNCCCTTTCTANNGANGGCANGCCCCCCGGGGATCCAAGGGGGCGACAATGGTGGCGCTTCAAAGGATTCGATGAATTCACGTGAATTCGTGCAATTCACATTACTTATCGCATTTCGCGTGCGATTCTTCATCGATGCCAGAACCAAGAGATCCGTTGTTGAAAGTTTTAACTATTAGATNGTCACNCAGACGACACTGACAATNCANAGTTGTGATTTCCCCCCGGA

> Sclerotinia homoeocarpa isolate 09 from OJN creeping bentgrass fall 2010; primers ITS/ITS2

CCCCGGAATCCAAGGGGCGCAATGTGCGCTTCAAAGATTCGATGATTCACGTGAATTCTGCAATTCACATTACTTATCGCATTTCGCGTGCGATTCTTCATCGATGCCAGAACCAAGAGATCCGTTGTTGAAAGTTTGAACTATTAGNNNGNCACNCAGACGACACTGACAATNCACANTNGTGATTTCCNCCCGGANGG

> Sclerotinia homoeocarpa isolate 10 from OJN creeping bentgrass fall 2010; primers ITS/ITS2

CCCCCGGAATCCAAGGGGCGCAATGGTGCGCTTCAAAGGATTCGATGATTCACTGAATTCTGCAATTCACATTACTTATCGCATTTCGCGTGCGATTCTTCATCGATGCCAGAACCAAGAGATCCGTTGTTGAAAGTTTTAACTATTAGANNGTCACNCAGACGACACTGACAATTCANANTTGTGATTTNCNNCCGGA

> Sclerotinia homoeocarpa isolate 11 from OJN creeping bentgrass fall 2010; primers ITS/ITS2

CCCCCGGAATCCAAGGGGCGCAATGTGCGCTTCAAAGATTCGATGATTCACTGAATTCTGCAATTCACATTACTTATCGCATTTCGCGTGCGATTCTTCATCGATGCCAGAACCAAGAGATCCGTTGTTGAAAGTTTTAACTATTAGNTNGTCACTCAGACGACACTGACAATTCANANTTGTGATTTNCCNCCGGA

> Sclerotinia homoeocarpa isolate 12 from OJN creeping bentgrass fall 2010; primers ITS/ITS2

CCCCGGAATCCAAGGGGCGCAATGTGCGCTTCAAAGATTCGATGATTCACTGAATTCTGCAATTCACATTACTTATCGCATTTCGCGTGCGATTCTTCATCGATGCCAGAACCAAGAGATCCGTTGTTGAAAGTTTTAACTATTAGNNNGTCACNCAGACGACACTGACAATTCANANTTGTGATTTNCCCCCGGAN

> Sclerotinia homoeocarpa isolate 13 from OJN creeping bentgrass fall 2010; primers ITS/ITS2

NCCTTACNAGCNCNGCANCCCCCGGGATCCAAGGGGCGCAATGGTGCGCTTCAAAGATTCGATGATTCACTGAATTCTGCAATTCACATTACTTATCGCATTTCGCTGCGATTCTTCATCGATGCCAGAACCAAGAGATCCGTTGTTGAAAGTTTTAACTATTAGNNNGTCACNCAGACGACACTGACAATNCANAGTTGTGATTTNCNCCCGGA

> Sclerotinia homoeocarpa isolate 14 from OJN creeping bentgrass fall 2010; primers ITS/ITS2

CCCCGGAATCCAAGGGGCGCAATGTGCGCTTCAAAGATTCGATGATTCACTGAATTCTGNNATTCACATTACTTATCGCATTTCGCGTGCGATTCTTCATCGATGCCAGAACCAAGAGATCCGTTGTNTGAAAGCTTTGTNACTATCAGNGNGNNANCCAGACNACACTGACNATNCACANTNGTGATCTCCNCCCGGAGG

> Sclerotinia homoeocarpa isolate 15 from OJN creeping bentgrass fall 2010; primers ITS/ITS2

CCCCCCGGGAATCCAAGGGGCGCAATGTGGCGCTTCAAAGGATTCGATGAATTCACTGAATTCTGCAATTCACATTACTTATCGCATTTCGCGTGCGATTCTTCATCGATGCCAGAACCAAGAGATCCGTTGTTGAAAGTTTGAACTATTAGNNNGNCACNCAGACGACACTGACAATNCANANTNGTGATTTNCNCCCGGAAG

> Sclerotinia homoeocarpa isolate 16 from OJN creeping bentgrass fall 2010; primers ITS/ITS2

CCCCCGGAATCCAAGGGGCGCAATGGTGCGCTTCAAAGATTCGATGATTCACTGAATTCTGCAATTCACATTACTTATCGCATTTCGCGTGCGATTCTTCATCGATGCCAGAACCAAGAGATCCGTTGTTGAAAGTTTTAACTATTAGNNNGTCACNCAGACGACACTGACAATTCANANTTGTGATTTNCNCCCGGAA

> Sclerotinia homoeocarpa isolate 17 from OJN creeping bentgrass fall 2010; primers ITS/ITS2

CCCCCGGAATCCAAGGGGCGCAATGTGCGCTTCAAAGATTCGATGATTCACTGAATTCTGCAATTCACATTACTTATCGCATTTCGCGTGCGATTCTTCATCGATGCCAGAACCAAGAGATCCGTTGTTGAAAGTTTGTAACTATTAGNNNGTCACNCAGACGACACTGACAATNCANANTTGTGATTT

> Sclerotinia homoeocarpa isolate 18 from OJN creeping bentgrass fall 2010; primers ITS/ITS2

CCCCCCGGAATCCAAGGGGCGCAATGTGCGCTTCAAAGATTCGATGATTCACTGAATTCTGCAATTCACATTACTTATCGCATTTCGCGTGCGATTCTTCATCGATGCCAGAACCAAGAGATCCGTTGTTGAAAGTTTTAACTATTAGNNNGTCACTCAGACGACACTGACAATTCAGAGTTGTGATTTTCCTCCGGA

> Sclerotinia homoeocarpa isolate 19 from OJN creeping bentgrass fall 2010; primers ITS/ITS2

CCCCCGGNATCCAAGGGGCGCAATGTGCGCTTCAAAGATTCGATGATTCACTGAATTCTGCAATTCACATTACTTATCGCATTTCGCGTGCGATTCTTCATCGATGCCAGAACCAAGAGATCCGTTGTTGAAAGTTTTAACTATTAGNNNGTCACTCAGACGACACTGACAATTCAGAGTTGTGATTTTCNNCCGGA

> Sclerotinia homoeocarpa isolate 20 from OJN creeping bentgrass fall 2010; primers ITS/ITS2

CCCCGGGAAATCCAAGGGGGCGCAATGTGCGCTTCAAAGGATTCGATGATTCACGTGAATTCGNGCAATTCACATTACTTATCGCATTTCGCGTGCGATTCTTCATCGATGCCAGAACCAAGAGATCCGTTGNTGAAAGTTTGAACTATTAGNNNGNCACNCAGACGACACTGACAATNCACANTNGTGATTTC

> Sclerotinia homoeocarpa isolate 21 from OJN creeping bentgrass fall 2010; primers ITS/ITS2

TCCCCCGGAATCCAAGGGGCGCAATGTGCGCTTCAAAGATTCGATGATTCACTGAATTCTGCAATTCACATTACTTATCGCATTTCGCTGCGATTCTTCATCGATGCCAGAACCAAGAGATCCGTTGTTGAAAGTTTGAACTATTAGNGNGNCANNCAGACGACACNGACAATNCANANTNGTGATTTNCNCCCGGA

> Sclerotinia homoeocarpa isolate 22 from OJN creeping bentgrass fall 2010; primers ITS/ITS2

CCCCCGGAATCCAAGGGGCGCAATGTGCGCTTCAAAGATTCGATGATTCACTGAATTCTGCAATTCACATTACTTATCGCATTTCGCGTGCGATTCTTCATCGATGCCAGAACCAAGAGATCCGTTGNTGAAAGTTTGAACTATTAGNNNGNCANNCAGACGACACTGACAATNCANANTTGTGATTTNCNCCCGGA

> Sclerotinia homoeocarpa isolate 23 from OJN creeping bentgrass fall 2010; primers ITS/ITS2

CANCCCCCCGGAATCCAAGGGGCGCAATGTGCGCTTCAAAGATTCGATGATTCACTGAATTCTGCNATTCACATTACTTATCGCATTTCGCGTGCGATTCTTCATCGATGCCAGAACCAAGAGATCCGTTGNTGAAAGTTTGAACTATTAGNNNGNCACNCAGACGACACNGACAATNCACANTNGTGATTTCCNCCCGGAG

> Sclerotinia homoeocarpa isolate 24 from OJN creeping bentgrass fall 2010; primers ITS/ITS2

TCANCCCCGGAATCCAAGGGGCGCAATGTGCGCTTCAAAGATTCGATGATTCACGTGAATTCTGCAATTCACATTACTTATCGCATTTCGCGTGCGATTCTTCATCGATGCCAGAACCAAGAGATCCGTTGTTGAAAGTTTTAACTATTAGANNGNCACNCAGACGACACTGACAATNCANANTTGTGATTTNCNCCCGGA

> Sclerotinia homoeocarpa isolate 25 from OJN creeping bentgrass fall 2010; primers ITS/ITS2

CCCCGGAATCCAAGGGGCGCAATGTGCGCTTCAAAGATTCGATGATTCACGTGAATTCTGCAATTCACATTACTTATCGCATTTCGCGTGCGATTCTTCATCGATGCCAGAACCAAGAGATCCGTTGTTGAAAGTTTGTAACTATTAGANNGTCACNCAGACGACACTGACAATTCANANTTGTGATTTNCCNCCGGA

> Sclerotinia homoeocarpa isolate 26 from OJN creeping bentgrass fall 2010; primers ITS/ITS2

CCCCGGAATCCAAGGGGCGCAATGTGCGCTTCAAAGATTCGATGATTCACTGAATTCTGCAATTCACATTACTTATCGCATTTCGCGTGCGATTCTTCATCGATGCCAGAACCAAGAGATCCGTTGNTGAAAGTTTGTAACTATTAGNNNGNCANNCAGACGACACNGACAATNCANANTNGTGATTTCCNCCCGGA

> Sclerotinia homoeocarpa isolate 27 from OJN creeping bentgrass fall 2010; primers ITS/ITS2

TCANCCCCCGGAAATCCAAGGGGCGCAATGTGCGCTTCAAAGATTCGATGATTCACTGAATTCTGCAATTCACATTACTTATCGCATTTCGCGTGCGATTCTTCATCGATGCCAGAACCAAGAGATCCGTTGNTGAAAGTTTGTAACTATTAGNGNGNCANNCAGACGACACNGACAATNCACANTNGTGNTTTCCNNCCGGAG

> Sclerotinia homoeocarpa isolate 28 from OJN creeping bentgrass fall 2010; primers ITS/ITS2

CCCCCGGGAATCCAAGGGGCGCAATGTGCGCTTCAAAGATTCGATGATTCACTGAATTCTGCAATTCACATTACTTATCGCATTTCGCGTGCGATTCTTCATCGATGCCAGAACCAAGAGATCCGTTGTTGAAAGTTTGTAACTATTAGNNNGNCACNCAGACGACACNGACAATNCANANTNGTGATTTNCNCCCGGA

> Sclerotinia homoeocarpa isolate 29 from OJN creeping bentgrass fall 2010; primers ITS/ITS2

TCANCCCCGGAATCCAAGGGGCGCAATGTGCGCTTCAAAGATTCGATGATTCACTGAATTCTGNAATTCACATTACTTATCGCATTTCGCGTGCGATTCTTCATCGATGCCAGAACCAAGAGATCCGTTGNTGAAAGTTTGAACTATTAGNNNGNCACNCAGACNACACTGACAATNCACANTNGTGATTTCCNCCCGGA

> Sclerotinia homoeocarpa isolate 30 from OJN creeping bentgrass fall 2010; primers ITS/ITS2

TCATCCCCCGGAATCCAAGGGGCGCAATGTGCGCTTCAAAGATTCGATGATTCACTGAATTCTGCAATTCACATTACTTATCGCATTTCGCGTGCGATTCTTCATCGATGCCAGAACCAAGAGATCCGTTGNTGAAAGTTTGAACTATTAGNGNGNCANNCAGACNACACTGACAATNCACANTNGTGATTTNCNCCCGGA

> Sclerotinia homoeocarpa isolate 31 from OJN creeping bentgrass fall 2010; primers ITS/ITS2

GCCCCCCGGNAATCCAAGGGGGCGCCAATGGTGGCGCTTCAAAGGATTCGGATGNATTCACGTGAAATTCGTGCAATTCACACTTACTTATCGCATTTCGCGTGCGATTCTTCATCGATGCCAGAACCAAGAGATCCGTTGNTGAAAGTTTTAACTATTAGNNNGNCACNCAGACGACACTGACAATNCANANTTGTGATTTNCNNCCGGA

> Sclerotinia homoeocarpa isolate 32 from OJN creeping bentgrass fall 2010; primers ITS/ITS2

TCANCCCCCGGAATCCAAGGGGCGCAATGTGCGTTCAAAGATTCGATGATTCACTGAATTCTGCAATTCACATTACTTATCGCATTTCGCGTGCGATTCTTCATCGATGCCAGAACCAAGAGATCCGTTGNTGAAAGTTTGAACTATCAGNGNGNCACNCAGACGACACTGACNATNCANANTNGTGATTTNCNNCCGGA

> Sclerotinia homoeocarpa isolate 33 from OJN creeping bentgrass fall 2010; primers ITS/ITS2

AGNCCCCGGGAAATCCAAGGGGCGCAATGTGACGCTTCAAAGGATTCGATGATTCACGTGAATTCGTGCAATTCACACTTACATTATCGCATTTCGCGTGCGATTCTTCATCGATGCCAGAACCAAGAGATCCGTTGTTGAAAGTTTGTAACTATTAGNNNGTCACNCAGACGACACTGACAATTCANANTNGTGATTTTCNNCCGGA

> Sclerotinia homoeocarpa isolate 34 from OJN creeping bentgrass fall 2010; primers ITS/ITS2

CCCCCGGAATCCAAGGGGCGCAATGGTGCGCTTCAAAGATTCGATGATTCACGTGAATTCNGCNATTCACATTACTTATCGCATTTCGCGTGCGATTCTTCATCGATGCCAGAACCAAGAGATCCGTTGNTGAAAGTTTGAACTATTAGNNNGNCACNCAGACGACACNGACAATNCANANTTGTGATTTNCNNCCGGA

> Sclerotinia homoeocarpa isolate 35 from OJN creeping bentgrass fall 2010; primers ITS/ITS2

CCCCGGAATCCAAGGGGCGCAATGGTGCGCTTCAAAGATTCGATGATTCACTGAATTCTGCAATTCACATTACTTATCGCATTTCGCGTGCGATTCTTCATCGATGCCAGAACCAAGAGATCCGTTGTTGAAAGTTTTAACTATTAGATNGTCACNCAGACGACACTGACAATTCANANTTGTGATTTNCCNCCGGA

> Sclerotinia homoeocarpa isolate 36 from OJN creeping bentgrass fall 2010; primers ITS/ITS2

CCCCCGGAAATCCAAGGGGCGCAATGTGCGCTTCAAAGATTCGATGATTCACTGAATTCTGCAATTCACATTACTTATCGCATTTCGCGTGCGATTCTTCATCGATGCCAGAACCAAGAGATCCGTTGTTGAAAGTTTGTAACTATTAGNNNGTCACNCAGACGACACTGACAATTCANANTTGTGATTTTCNNCCGGA

>Sclerotinia homoeocarpa isolate 37 from OJN creeping bentgrass fall 2010; primers ITS/ITS2

TCAGNCNCCCCGGAAATCCAAGGGGGCGCAATGTGACGCTTCAAAGGATTCGATGATTCACGTGAATTCGTGCAATTCACATTACTTATCGCATTTCGCGTGCGATTCTTCATCGATGCCAGAACCAAGAGATCCGTTGTTGAAAGTTTGTAACTATTAG

>Sclerotinia homoeocarpa isolate 38 from OJN creeping bentgrass spring 2011; primers ITS/ITS2

NCANATCGTNTNANANANNGNNNAGTTTACTTTAGATAGTTACNTCTGANGACACTGGACAATTCTGAGCTTGTGTATTTTCCTCCNGCCAGGCGGACATCTCCGGCCCCGGAGGGNGCCTGAGGNATGTNCCCNGAAGGGTCNAGACANCCTGCCNAAGCATCATGGTANANATACACAAAGNTTGNAGGTCTACCCNTGAGGGCGTGAACTCGGAAATGATCCTTCCNCAAGTTCANCTACNGAAG

**PCR confirmation of *Sclerotinia homoeocarpa* isolate from creeping bentgrass cv. Shark seed**

>Sclerotinia homoeocarpa isolate Shark from Shark seed lot 1; primers ITS1/ITS2

GTTCAGAGCTTGGTGTATTTTCCTCCGGCCAGGCGAACATCTCCGGCCCCGGTAGGGCGCTGAGGCATGTCCCCGGAAGGGTCAAGCAGCCTGCCAAAGCAACATGGTANAGATACACNANGGTTGGAGGTCTACCCGTGAGGGCGTGAACTCGGTAATGATCCTTCCGCANGTCCACCTACNGAAG

**Sequences of nested PCR products from molecular detection of *Sclerotinia homoeocarpa* in creeping bentgrass seed lots**

>Sclerotinia homoeocarpa_EF1-alpha_Shark650_1; primers EF1α_F/EF1α_R

TCGGTATGACTTCTCCACCTTTCTCTTGCTATCTTTTCCCGTCCTTCTCATCGAGATCAG TGTCTGCGATCTTGGTGCTGATGGATTTATCGGGTTGCGTTTTCTCTCATGCGCGGAGCA TACATCCGAATTCTCAACCCTTTGAACATTACCACATTGCCTTTCCAGAATCCCTTTGCT AACCCGTTAATAGGAAGCCAAGGAGATGGGGAAAGGGTTCA

>Sclerotinia homoeocarpa_EF1-alpha_Shark650_2; primers EF1α_F/EF1α_R

TCGGTATGACTTTCTCCACCTTTCTCTTGCTATCTTTTCCCGTCCTTCTCATCGAGATCA GTGTCTGCGATCTTGGTGCTGATGGATTTATCGGGGTTGCGTTTTCTCTCATGCGCGGAG CATACATCCGAATTCTCAACCCTTTGAACATTACCACATTGCCTTTCCAGAATCCCTTTG CTAACCCGTTAATAGGAAGCCAAGGAGATGGGAAAGGGTTCAA

>Sclerotinia homoeocarpa_EF1-alpha_Shark7881_1; primers EF1α_F/EF1α_R

TCTCGGTATGACTTCTCCACCTTTCTCTTGCTATCTTTTCCCGTCCTTCTCATCGAGATC AGTGTCTGCGATCTTGGTGCTGATGGATTTATCGGGTTGCGTTTTCTCTCATGCGCGGAG CATACATCCGAATTCTCAACCCTTTGAACATTACCACATTGCCTTTCCAGAATCCCTTTG CTAACCCGTTAATAGGAAGCCAAGGAGATGGGAAAGGGTTCAA

>Sclerotinia homoeocarpa_EF1-alpha_Shark7881_2; primers EF1α_F/EF1α_R

TCGGTATGACTTTCTCCACCTTTCTCTTGCTATCTTTTCCCGTCCTTCTCATCGAGATCA GTGTCTGCGATCTTGGTGCTGATGGATTTATCGGGTTGCGTTTTCTCTCATGCGCGGAGC ATACATCCGAATTCTCAACCCTTTGAACATTACCACATTGCCTTTCCAGAATCCCTTTGC TAACCCGTTAATAGGAAGCCAAGGAGATGGGGAAAGGGTTCAA
